# Non-targeted metabolomics of moldy wheat by ultra-performance liquid chromatography – quadrupole time-of-flight mass spectrometry

**DOI:** 10.3389/fmicb.2023.1136516

**Published:** 2023-04-06

**Authors:** Boyan Gao, Weiying Lu, Mengchu Jin, Yumei Chen

**Affiliations:** Department of Food Science & Engineering, School of Agriculture and Biology, Institute of Food and Nutraceutical Science, Shanghai Jiao Tong University, Shanghai, China

**Keywords:** wheat, mildew, non-targeted detection, UPLC-QTOF-MS, metabolomics

## Abstract

**Introduction:**

As one of the staple foods for the world’s major populations, the safety of wheat is critical in ensuring people’s wellbeing. However, mildew is one of the prevalent safety issues that threatens the quality of wheat during growth, production, and storage. Due to the complex nature of the microbial metabolites, the rapid identification of moldy wheat is challenging.

**Methods:**

In this research, identification of moldy wheat samples was studied using ultra-performance liquid chromatography - quadrupole time-of-flight mass spectrometry (UPLC-QTOF-MS) coupled with chemometrics. The non-targeted PCA model for identifying moldy wheat from normal wheat was established by using previously established compounds database of authentic wheat samples. The partial least squares-discriminant analysis (PLS-DA) was performed.

**Results and discussion:**

By optimizing the model parameters, correct discrimination of the moldy wheat as low as 5% (w/w) adulteration level could be achieved. Differential biomarkers unique to moldy wheat were also extracted to identify between the moldy and authentic wheat samples. The results demonstrated that the chemical information of wheat combined with the existing PCA model could efficiently discriminate between the constructed moldy wheat samples. The study offered an effective method toward screening wheat safety.

## 1. Introduction

Wheat is one of the major grains in the world. However, the frequent occurrence of wheat safety issues not poses a threat to the protection of food nutrition and health of the public, but greatly restricts the exportation of wheat, causing economic losses (Dong et al., 2012). Risks of food contamination exist at every stage of food production and processing, including biological, physical, and chemical pollution. During planting, harvesting, drying, milling, and transportation, the surface of wheat grain may be contaminated by harmful microorganisms such as typically fungi (Li et al., 2003). Additionally, improper storage of wheat grain may cause excessive reproduction of fungi, triggering safety incidents and threatening the safety of wheat and related products (Femenias et al., 2020). Fungi may possess a series of strongly toxic metabolites (Sabillon and Bianchini, 2016). The common fungi in contaminated wheat are *penicillium* and *aspergillus*, with the presence of typical mycotoxins such as aflatoxin, deoxynivalenol, ochratoxin, vomitoxin (Wei et al., 2019). Mycotoxins are small but stable toxins, they are difficult to remove and easily enters the food chain by direct human consumption. These mycotoxins bring a huge threat to public wellbeing. Generally, different types of mycotoxins are produced according to different fungal species. For example, deoxynivalenol, also referred to as vomitoxin, is a toxin produced by *Fusarium graminearum*, commonly found in wheat and wheat products. Deoxynivalenol is a common hepatotoxic toxin in wheat with different approaches (Li et al., 2016; Vidal et al., 2017; Cirio et al., 2019), with acute vomiting and may even be life-threatening (Gu et al., 2019) symptoms after consumption. Therefore, the development of efficient and feasible early-warning approaches to ensure the safety of wheat and its products is required.

Metabolomics can depict actual physiological metabolic state of the sample by comprehensively profiling its metabolites (Rana et al., 2020). Microbiological contamination can have corresponding impacts on a specific food system (Creydt and Fischer, 2018). Especially, mycotoxins produced by fungi have a great threat to life and health and have always been one of the main factors for wheat safety in fungal-contaminated wheat (Ramadhaningtyas et al., 2017). As a result, metabolomics in food products, usually be referred to as foodomics, can be a suitable approach for a series of food quality identification applications, including detecting possible microbial contamination (Cifuentes, 2009; Herrero et al., 2012).

The complexity of the compounds brought by the metabolomics made the identification of contamination a challenging task. Therefore, it is common to apply techniques such as liquid chromatography-mass spectrometry (LCMS) with high sensitivity and selectivity over a wide range of concentrations. Keskin et al. quantified mycotoxins in bee products of Turkey (KesKin and Eyupoglu, 2023). Sulyok et al. quantified more than 500 mycotoxins by liquid chromatography coupled with tandem mass spectrometry (LC–MS/MS) on nuts, wheats and raisins (Sulyok et al., 2020). However, the targeted approach requires specific standard for every analyte compound to establish calibration curves prior to sample analysis, which is time-and cost-consuming. The validation of matrix effects from complicated food matrices is also needed by proper reference materials (Gab-Allah et al., 2023). Additionally, the targeted analysis is not suitable when the standard is not commercially available. The situation is even more difficult when the exogenous sources of microorganisms is unknown. The emergence of non-targeted detection strategy offers new opportunities for food safety and screening of samples, as well as discovering possible markers (Yu et al., 2016; Gao et al., 2019; Sun et al., 2021). Non-targeted detection focuses on all the chemical information in the entire sample being analyzed, rather than quantitively studying limited number of markers of interest. In a broader sense, non-targeted detection mainly refers to screening unknown chemical substances and identifying chemical components in mixed systems according to omics methods (Kunzelmann et al., 2018), while non-targeted detection in the narrow sense can be understood as relying on the established chemical hazard factor database to identify the correctness of unknown samples and filter out intentional adulteration or contaminants (Hakme et al., 2017). Different from the targeted approaches, non-targeted methods have been widely practiced in detecting food fraud and quality in recent years. It has been proven to be capable of clearly differentiate a large set of samples and of efficiently identify abnormal samples without standards. Combining with chemometric data processing, non-targeted detection aims to find abnormal signals in the sample without *a priori* knowledge. Consequently, it is suitable for rapid screening of food contamination, because prior investigation of compounds is not required.

This study attempts to rapidly identify authentic and moldy wheat, and to achieve non-targeted screening model of moldy wheat without any specific identification of spectral peaks. In this work, the moldy wheat is simulated and analyzed by ultrahigh performance-liquid chromatography-quadrupole time-of-flight-mass spectrometry (UPLC-QTOF-MS). The entire LCMS profile is then directly applied based on non-targeted metabolomics to achieve chemometrics analyses. Distinguishing abnormal samples spiked with different proportions of moldy wheat from normal wheat samples, and the parameters of the non-targeted detection model were further optimized to achieve a good screening effect of abnormal wheat samples. Finally, the differences in the chemical composition of small molecules between the mildew-contaminated wheat and the authentic wheat samples were obtained, thus discovering differential compounds in wheat that caused mildew. This work can provide valuable insight toward non-targeted detection without the need of standardized compound information.

## 2. Materials and methods

### 2.1. Materials and chemical reagents

Fourteen wheat grains were gifted by local breeding institutes in 2018 and stored at –4°C before analyses. The samples were from eight provinces in China, including Anhui, Fujian, Guizhou, Guangdong, Hubei, Henan, Gansu, and Shaanxi. Two samples were collected for each province, except that one sample were collected from Guizhou and Guangdong province. Each sample were prepared and tested in triplicates. Methanol, acetonitrile, isopropanol, and formic acid were in LCMS grade and purchased from Merck KGaA (Darmstadt, Germany). HPLC grade dichloromethane used for extraction was purchased from Sigma-Aldrich (St. Louis, MO, United States). Water was purified by a Milli-Q 10 ultrapure water system (Millipore Laboratory, Bedford, MA, United States).

### 2.2. Sample preprocessing and extraction

Wheat samples were milled by an A11 laboratory grinder (IKA, Staufen, Baden-Württemberg, Germany). A total of 2 g of each sample was mixed to make quality control (QC) sample. The mildew conditions of wheat were performed according to the previous work (He et al., 2019), with wheat severely mildewed in a short time. Five grams of each sample was placed in a 50 mL Petri dish. Then the samples were incubated at 40°C and 50% humidity for 5 d. Afterward, the wheat samples were dried at 40°C and grounded into powder. Fifty milligrams of powder made from each sample were weighed and mixed to obtain mixed QC. To simulate different degree of mildew of authentic wheat, the mixed QC samples were mixed into normal QC wheat samples at 5, 10, 20, 30, 40, 50, and 80% (w/w) levels.

Sample extraction was adapted from the previous work (Righetti et al., 2016; Jin et al., 2021). A total of 50 mg wheat flour was accurately weighted and extracted by 1.5 mL 7:3 ethanol/dichloromethane (v/v) in a 2 mL polypropylene centrifugation tube. After vortexing for 60 s, the mixtures were placed in an ultrasonic water bath (Hechuang Ultrasonic, Shanghai, China) with the power consumption at 400 W and ambient temperature for 30 min. The analyte was then centrifuged at 13,000 *g* and kept at 4°C for 10 min. Afterwards, the supernatants were extracted and filtered through a 0.22 μm syringe filter for analysis.

### 2.3. UPLC-QTOF-MS analyses

The analyses were carried out by an ACQUITY ultra-performance liquid chromatograph hyphenated with a Xevo G2 quadrupole time-of-flight mass spectrometer (UPLC-QTOF-MS) (Waters, Milford, MA, United States). A Waters Acquity UPLC BEH C18 column (2.1 × 150 mm i.d.; 1.7 μm) with the column temperature maintained at 45°C were applied during analysis. The gradient elution was carried out at a constant flow rate at 0.3 mL/min with 0.1% formic acid (solvent A) and 0.1% formic acid in 4:6 isopropanol/acetonitrile (v/v, solvent B). One aliquot of 2 μl analytes were injected. The linear gradient was performed as follows: 0–1 min, 10–50% B; 1–2 min, 50% B; 2–5 min, 50–80% B; 5–8 min, 80–90% B; 8–14 min, 90–91% B; 14–16 min, 91–95% B; 16–18 min, 95–96.5% B; 18–22 min, 96.5–97% B; 22–25 min, 97–100%; 25–28 min, 100% B; 28–28.1 min, 100–10% B; 28.1–30 min, 10% B. The electrospray ionization (ESI) source was applied and set in the positive ionization mode, with the source and desolvation temperature at 120°C and 450°C. The voltages for capillary, sampling and extraction cones were 2.5 kV, 40 V, and 4.0 V, respectively. The flow rates of cone and desolvation gasses were 100 and 800 L/h, respectively. The mass-to-charge ratio was 100–1,200 m/z with the collision energy of 6 eV in MS^1^ function, while the fragment signals were in the same m/z range in MS^2^ scan with the collision energy ramping from 20–35 eV.

### 2.4. Data processing

A customized in-house workflow is implemented to achieve a fully-automated chemometric processing for LCMS data set. Specifically, the acquisition of the raw data was performed using MassLynx version 4.2 (Waters). Raw spectra were transformed to mzXML format using “msconvert” tool in ProteoWizard software library (version 3.0.22099.89, 64-bit, Center for Applied Molecular Medicine, University of Southern California, Los Angeles, CA, United States). The LCMS peaks were further identified and extracted by pyOpenMS module (version 2.7.0) running on Python version 3.9 (Python Software Foundation, http://www.python.org). pyOpenMS is an interface for the OpenMS library for computational mass spectrometry (Röst et al., 2016). All mass spectra were extracted at the retention time tolerance of 0.1 min and the 500 most intensive peaks were extracted in MS^1^ function of each LCMS spectrum. The peaks with mass error less than 10 ppm were considered to be the same compound across different LCMS runs. The intensity for each exported mass spectrum is summed to represent the intensity of the corresponding compound in a mass spectrum, rather than the intensities of every fragments from a series of peaks in each spectrum. Finally, 11,519 peaks were subjected to chemometrics modelling.

The list of identified peaks was then delivered to MATLAB R2021b (MathWorks, Natick, MA) for chemometrics modelling, including PCA and PLS-DA analysis. PCA and PLS-DA were performed using the built-in functions from the Statistics Toolbox in MATLAB. Autoscaling, i.e., mean-centering and scaling to unit variance were applied before PCA. All other MATLAB routines were written in-house. Their application performances for non-targeted screening of wheat were evaluated and compared. The PLS-DA model was used to distinguish the normal wheat samples from the contaminated wheat samples in a supervised approach.

## 3. Results and discussion

### 3.1. Morphological characteristics of moldy wheats

**Figure 1:**
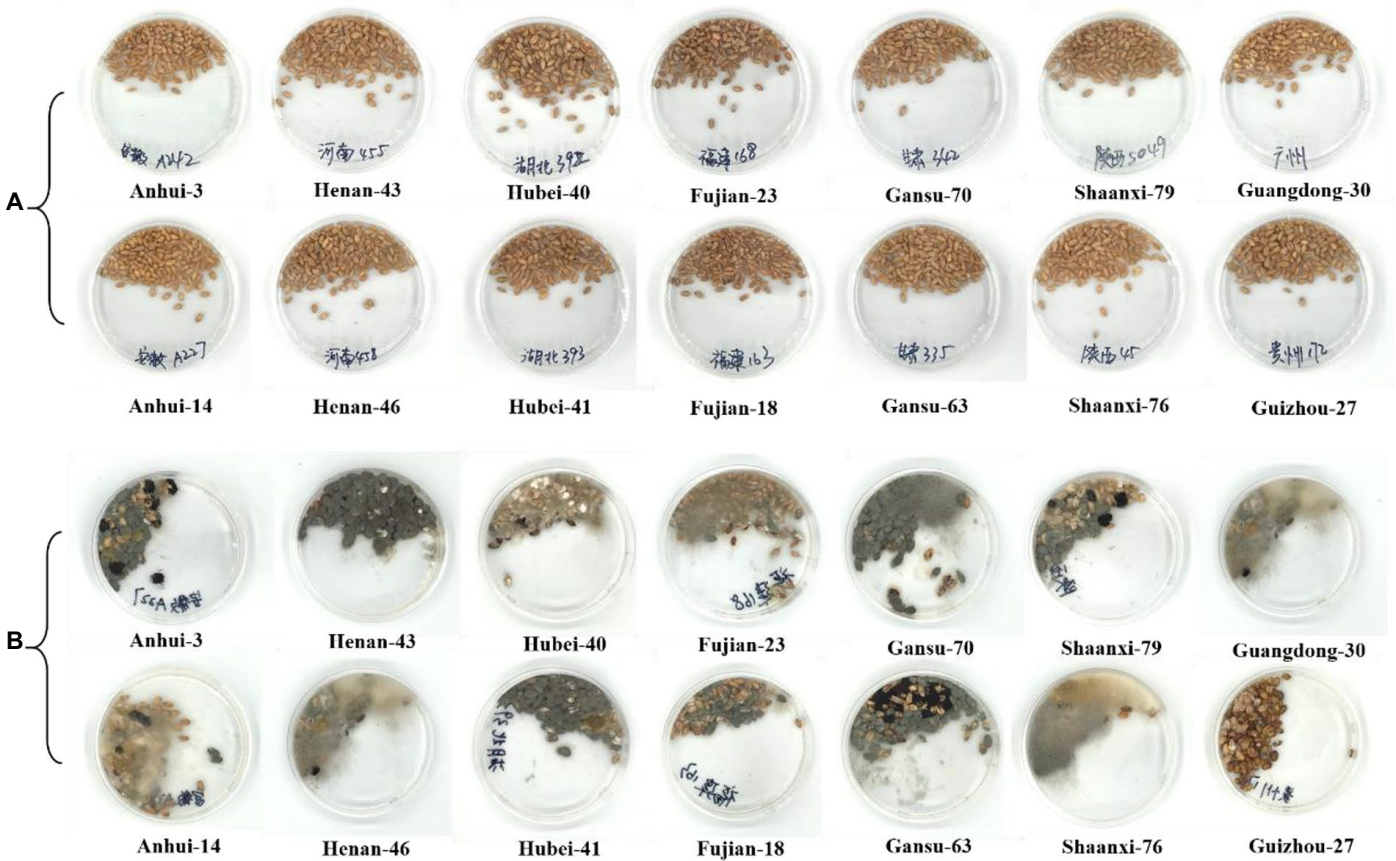
Appearances of wheat samples before and after mildew treatment. **(A)** Normal wheat samples; **(B)** Wheat samples after mildew treatments.

The morphology characteristics of wheats was first observed for preliminary visual confirmation of molds. The wheat samples before and after mildew treatment are shown in Figure 1. It is speculated that there may have been mold contamination. Overall, the molds growing on the surface of the abnormal samples by visual inspection were all fluffy objects with gray-green, yellow-green, black, or white colors. The possible molds in wheat includes *Aspergillus niger*, *Aspergillus oryzae*, *Penicillium*, etc., all with different visual characteristics (He et al., 2019). The result is relevant with previous studies which reported wheat is more prone to mildew under high temperature and humidity during storage (Huang et al., 2010; Zhou et al., 2010). It is also worth noting that samples had an inconsistent appearance, indicating a different mildew degree. For instance, it is manifested that sample No. 27 from Guizhou was less mildew than other samples in morphological observations. Due that the differences in the degree of mildew and in the mold species, visual confirmation is not suitable for actual screening application. As a consequence, non-targeted screening by mass spectrometric fingerprints was carried out further.

### 3.2. Unsupervised non-targeted screening of wheats by PCA

**Figure 2:**
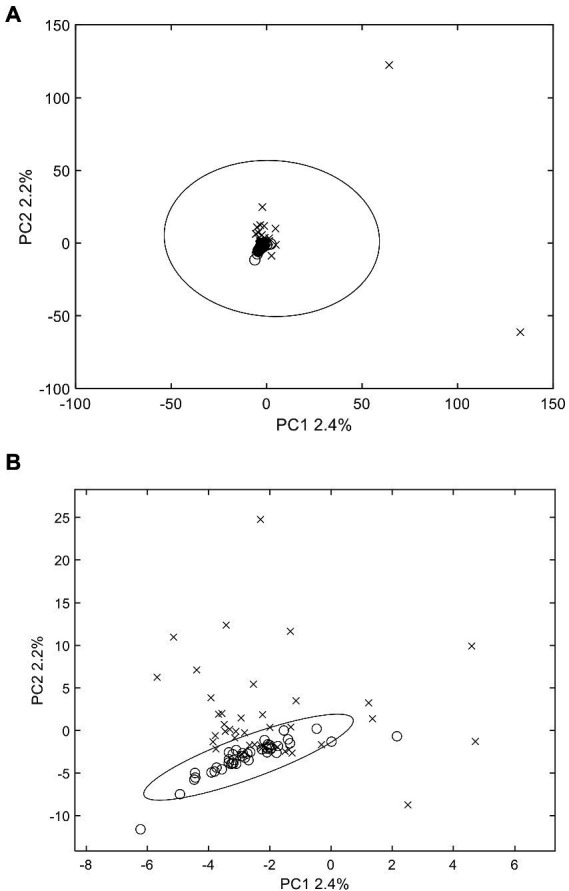
PCA scores plot of wheat samples. **(A)** includes all samples and **(B)** is the zoomed view of the central area for better visual presentation. The 95% Hotelling’s T^2^ confidence ellipse is plotted for each class. o: normal samples; x: moldy samples.

PCA scores plot was applied to examine whether there were differences between MS fingerprints of normal wheats and their moldy counterparts. PCA can indicate the clustering situation of all the original information (Cordewener et al., 2009). The results indicated that PCA, as an unsupervised approach, could generally provide efficient classification characteristics for the moldy wheat samples (Figure 2). Specially, the PCA scores plot of the two largest principal components indicated that normal wheat samples are all clustered in the center of the scores plot. Most of the normal samples were inside the 95% confidence ellipse, except two normal wheat samples from Anhui and Guizhou falls outside. The moldy wheat samples were widely apart to each other compared to their normal counterparts. Most of the moldy samples were outside of the 95% confidence ellipse of the normal samples, but there were still several moldy samples fell within the confidence ellipse that might be misidentified as normal samples according to PCA scores alone. These moldy samples were misclassified as normal, possibly due that the degree of mildew such as Guizhou-27 is inconsistent according to previous morphological studies, leading to a mild difference in chemical composition compared with the normal samples. Although separation can be observed to some extent, overall, the confidence ellipse of moldy wheat is far larger than that of the normal samples and completely wrapped around the normal samples, indicating a statically unsuccessful separation of the two classes. Two samples were especially apart from all other samples, i.e., two samples from Hubei and Henan. The inconsistency of the degree of separation is possibly due to the degree of mildew which needs to investigate further. There still exist false results in the current model. PCA is an unsupervised pattern recognition method, i.e., when modelling by PCA the classification information between the model samples is not given in advance. Therefore, unsupervised pattern recognition may be weak in terms of the classification power for the current research. Additionally, PCA scores plot only generates a brief overview of the samples, but it could not provide direct and fully-automated information about contamination. Therefore, it was necessary to further evaluate by other chemometrics models to achieve better performance. The research of differential substances between moldy wheat and normal wheat requires supervised approach. Consequently, PLS-DA was tested further for predicting the presence of moldy wheat.

### 3.3. Supervised evaluation of the untargeted screening model for abnormal wheats

**Figure 3:**
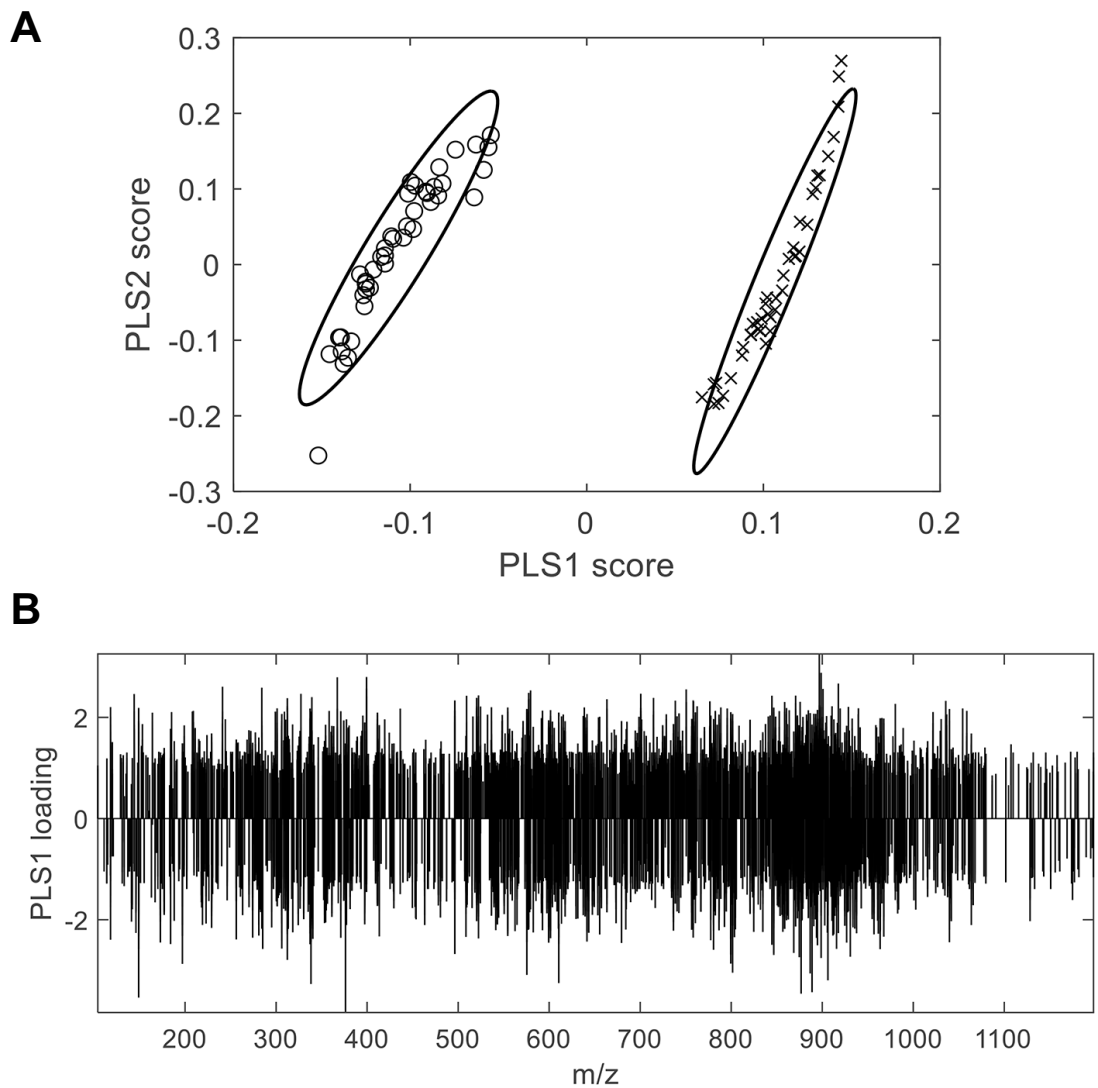
PLS-DA X-scores plot **(A)** and loading plot **(B)** of abnormal wheat screening. o: normal samples; x: moldy samples.

The supervised models that highlight the differences between samples are applicable for ascertaining differential substances in moldy and normal wheats, as grouping information was given prior to modelling. In the previous section, PCA did not completely separate the two class of samples. In comparison, the PLS-DA algorithm constructs a regression model and removes interference information through orthogonal correction. Figure 3 depicted the PLS-DA scores plot of authentic and moldy wheat. It was clearly observed from Figure 3A that there was significant discrimination of the two types of wheat samples, with each sample group clustered closely. All suspected samples were outside the 95% confidence interval, indicating the significant differences in the chemical compositions of the two types of wheat. It is worth to mention that the degree of separation on only the largest latent variable (PLS1) already yielded the good separation. A further investigation of a 10-fold cross validation yielded 95.24% correct prediction with only 1 latent variable for PLS modelling. Based on the Occam’s razor rule, further PLS-DA studies in this work will only use only one latent variable. The results demonstrated that the extraction method of wheat samples and the non-targeted screening model constructed in this study could correctly identify mildew-contaminated abnormal wheat under a supervised learning model. The current methods can attain complete screening of wheat with different degrees of mildew constructed by simulation. Therefore, PLS-DA with LCMS profiling could potentially be used to detect the molds at levels 5% and above.

Besides the scores plot, the supervised PLS-DA model seek the variables in the loading plot and obtains differential compounds between the two types of wheat samples. The loading plot of PLS-DA (Figure 3B) displayed the potential chemical information that may differentiate the two types of samples. Although no clear tendency can be discovered between the overall compound m/z and the loading, some useful information still can be observed from the loading. First, differential compounds that had a major impact on the classification performance of the model were from both the normal and moldy wheat samples, with corresponding negative and positive loadings. The number of compounds with positive and negative loadings were close, indicating the balanced ability for this model to extract marker information from both normal and moldy wheats. Second, it can be seen that a large number of unknown new abnormal compounds appeared in the mildew process, probably due to a mold-induced change in endogenous substances in wheat or a mold metabolite. Meanwhile, the inspection of raw mass spectra confirmed that most of the differential compounds screened out were not present in the normal samples, suggesting the existence of a large amount of abnormal information.

The representative marker compounds may present useful information for potential marker discovery. Table 1 listed potential discriminant marker for both endogenous and exogenous chemicals, respectively. It can be observed that of all 62 typical endogenous compounds identified in our previous work (Jin et al., 2021), 59 were successfully detected in the present model, indicating a good repeatability of the non-targeted mass spectral fingerprints. Thirty-four endogenous markers were with negative loadings, most of them were triacylglycerols (TG) such as TG (16:0/18:1/18:1) and TG (16:0/16:0/16:0), indicating a probable loss of nutrition due to the mildew process. However, 25 other compounds, especially lysophosphatidylcholine (16:0) showed positive loadings, indicating a potential increase in the corresponding substances in moldy samples. Overall, the results showed that the endogenous compounds were significantly altered due to the mildew process. However, there is no clear tendencies for each individual compound, which showed that the effect of microbial metabolism is complicated.

**Table 1:** Potential endogenous markers.

No.	RT^*^ (min)	Compound name^*^	Chemical formula	Adduct	Calc. m/z	Exptl. m/z^*^	PLS1 loading
1	4.79	Linolenic acid	C_18_H_30_O_2_	H	279.2324	279.2324	−1.313
2	5.7	Lyso PC (18:3)	C_26_H_48_NO_7_P	H	518.3247	518.3246	−0.933
3	6.21	Lyso PC (18:2)	C_26_H_50_NO_7_P	H	520.3403	520.3403	1.503
4	6.57	Linoleic acid	C_18_H_32_O_2_	H-H_2_O	263.2375	263.2375	1.208
5	6.73	Lyso PC (16:0)	C_24_H_50_NO_7_P	H	496.3403	496.3403	2.028
6	6.85	Lyso PC (18:1)	C_26_H_52_NO_7_P	H	522.3560	522.356	0.205
7	7.24	Oleic acid	C_18_H_34_O_2_	H-H_2_O	265.2531	265.2529 (1)	0.982
8	7.71	Lyso PC (18:0)	C_26_H_54_NO_7_P	H	524.3716	524.3716	−1.504
9	9.59	13-Docosenamide	C_22_H_43_NO	H	338.3423	338.3423	−1.264
10	10.53	DGDG (18:3/18:2)	C_51_H_86_O_15_	Na	961.5864	961.5864	−0.049
11	10.56	PC (18:2/18:3)	C_44_H_78_NO_8_P	H	780.5543	780.5543	−0.812
12	10.9	β-Sitosterol	C_29_H_50_O	H-H_2_O	397.3834	397.3834	1.231
13	11.21	MGDG (18:3/18:2)	C_45_H_76_O_10_	Na	799.5336	799.5336	1.257
14	11.54	PC (18:2/18:2)	C_44_H_80_NO_8_P	H	782.5700	782.5700	−0.367
15	11.72	PC (16:0/18:3)	C_42_H_78_NO_8_P	H	756.5543	756.5543	−1.308
16	11.72	DGDG (16:0/18:3)	C_49_H_86_O_15_	Na	937.5864	937.5860	−1.599
17	11.99	PE (18:2/18:2)	C_41_H_74_NO_8_P	H	740.5230	740.5230	0.748
18	12.36	MGDG (18:2/18:2)	C_45_H_78_O_10_	Na	801.5493	801.5493	−1.437
19	12.88	DGDG (18:2/18:1)	C_51_H_90_O_15_	Na	965.6177	965.6177	−0.905
20	12.94	PC (18:2/18:1)	C_44_H_82_NO_8_P	H	784.5856	784.5856	1.569
21	13.00	DGDG (16:0/18:2)	C_49_H_88_O_15_	Na	939.6021	939.6019	−0.519
22	13.04	PC (16:0/18:2)	C_42_H_80_NO_8_P	H	758.5700	758.5700	−0.274
23	13.68	PE (16:0/18:3)	C_39_H_74_NO_8_P	H	716.5230	716.5228	1.308
24	14.02	MGDG (18:2/18:1)	C_45_H_80_O_10_	Na	803.5649	803.5649	0.640
25	14.09	DG (18:2/18:2)	C_39_H_68_O_5_	Na	639.4964	639.4964	1.536
26	14.14	MGDG (18:2/16:0)	C_43_H_78_O_10_	Na	777.5493	777.5493	−2.008
27	14.82	Cer (d18:0/16:0)	C_34_H_69_NO_3_	Na	562.5175	562.5175	0.952
28	14.86	PC (18:1/18:1)	C_44_H_84_NO_8_P	H	786.6013	786.6013	−1.777
29	14.88	DGDG (18:1/16:0)	C_49_H_90_O_15_	Na	941.6177	941.6213 (4)	1.803
30	14.98	PC (16:0/18:1)	C_42_H_82_NO_8_P	H	760.5856	760.5856	−0.808
31	15.08	PC (16:0/16:0)	C_40_H_80_NO_8_P	H	734.5700	734.5700	0.160
32	15.13	DGDG (18:0/18:2)	C_51_H_92_O_15_	Na	967.6334	967.6333	−1.023
33	15.84	DG (18:1/18:2)	C_39_H_70_O_5_	H-H_2_O	601.5196	601.5196	−1.500
34	15.91	DG (18:2/16:0)	C_37_H_68_O_5_	H-H_2_O	575.5039	575.5039	0.187
35	16.21	DG (18:2/18:1)	C_39_H_70_O_5_	Na	641.5121	641.5121	0.514
36	17.57	DG (18:1/18:1)	C_39_H_72_O_5_	H-H_2_O	603.5352	603.5352	0.299
37	17.64	DG (16:0/18:1)	C_37_H_70_O_5_	H-H_2_O	577.5196	577.5196	1.208
38	18.90	TG (18:3/18:2/18:2)	C_57_H_96_O_6_	H	877.7285	877.7285	−1.994
39	20.17	TG (18:2/18:2/16:1)	C_55_H_96_O_6_	H	853.7285	853.7285	1.085
40	20.59	TG (18:2/18:3/18:3)	C_57_H_94_O_6_	NH_4_	892.7394	892.7393	−1.736
41	21.43	TG (16:1/18:1/18:2)	C_55_H_98_O_6_	H	855.7442	855.7431 (1)	0.227
42	21.87	TG (18:3/18:2/18:2)	C_57_H_96_O_6_	NH_4_	894.7551	894.7549	0.169
43	22.27	TG (16:0/18:3/18:3)	C_55_H_94_O_6_	NH_4_	868.7394	868.7395	1.308
44	23.42	TG (18:3/18:1/18:2)	C_57_H_98_O_6_	NH_4_	896.7707	896.7706	−1.231
45	23.87	TG (18:3/18:2/16:0)	C_55_H_96_O_6_	NH_4_	870.7551	870.7551	1.287
46	25.12	TG (18:1/18:2/18:2)	C_57_H_100_O_6_	NH_4_	898.7864	898.7864	−0.604
47	25.37	TG (16:0/18:2/18:2)	C_55_H_98_O_6_	NH_4_	872.7707	872.7709	−0.933
48	26.46	TG (20:1/18:2/18:2)	C_59_H_104_O_6_	NH_4_	926.8177	926.8176	−1.285
49	26.59	TG (18:2/18:1/18:1)	C_57_H_102_O_6_	NH_4_	900.8020	900.8019	−0.746
50	26.81	TG (18:2/18:1/16:0)	C_55_H_100_O_6_	NH_4_	874.7864	874.7864	−0.148
51	27.00	TG (18:2/16:0/16:0)	C_53_H_98_O_6_	NH_4_	848.7707	848.7707	−0.751
52	27.66	TG (18:2/22:1/18:2)	C_61_H_108_O_6_	NH_4_	954.8490	954.8489	−0.937
53	27.79	TG (18:2/18:1/20:1)	C_59_H_106_O_6_	NH_4_	928.8333	928.8334	−0.641
54	27.97	TG (20:1/16:1/18:1)	C_57_H_104_O_6_	NH_4_	902.8177	902.8176	0.129
55	28.23	TG (16:0/18:1/18:1)	C_55_H_102_O_6_	NH_4_	876.8020	876.8020	−1.668
56	28.49	TG (18:1/16:0/16:0)	C_53_H_100_O_6_	NH_4_	850.7864	850.7863	−1.425
57	28.84	TG (16:0/16:0/16:0)	C_51_H_98_O_6_	NH_4_	824.7707	824.7707	−1.512
58	29.04	TG (24:1/18:2/18:2)	C_63_H_112_O_6_	NH_4_	982.8803	982.8803	−1.356
59	29.38	TG (18:1/18:1/20:1)	C_59_H_108_O_6_	NH_4_	930.8490	930.8489	−1.288

* RT, retention time; TG, triacylglycerols; DG, diglyceride; PC, phosphatidylcholine; Lyso PC, lysophosphatidylcholines; DGDG, digalactosyldiacylglycerol; MGDG, monogalactosyldiacylglycerol; Cer, ceramide; Calc. m/z: calculated m/z; Exptl. m/z: Experimental m/z. When the mass error is greater than 1 ppm, the mass errors in ppm is indicated in parentheses following the experimental m/z.

**Figure 4:**
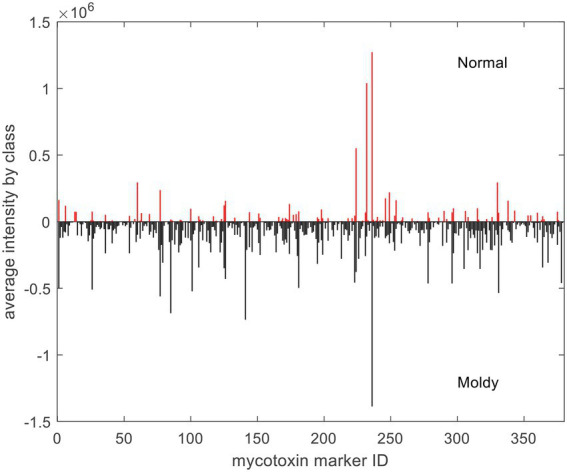
Averaged intensities of identified mycotoxin markers by different class of samples. For demonstration purpose, negative intensities were plotted for moldy samples.

**Figure 5:**
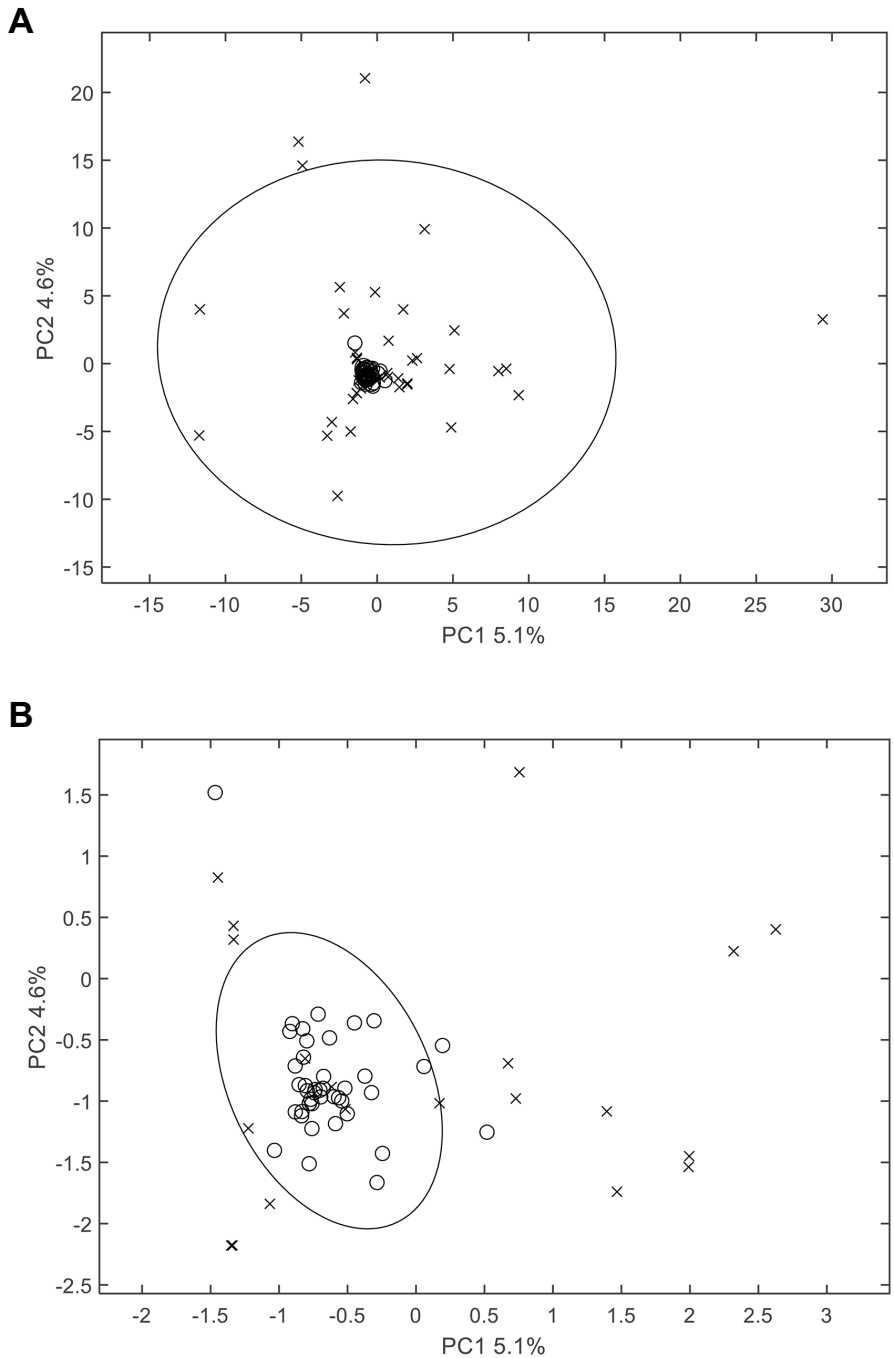
PCA scores plot of wheat samples by mycotoxin markers only. **(A)** includes all samples and **(B)** is the zoomed view of the central area for better visual presentation. The 95% Hotelling’s T^2^ confidence ellipse is plotted for each class. o: normal samples; x: moldy samples.

With respect to exogenous contaminations, compounds were searched by an in-house mycotoxin database built according to a previous work (Sulyok et al., 2020). A total of 379 mycotoxin markers were tentatively identified (Supplementary Table S1). Among all the compounds, 353 were with positive PLS loadings, which suggested reasonable suspicion for the presence of various mycotoxins in moldy wheats. For example, among the differential compounds identified in the current study, compound m/z = 315.0868 [M + H]^+^ (PLS1 loading = 1.792) was consistent with the molecular formula of aflatoxin B2 with a mass error of 0 ppm, and only recognized in moldy wheat samples. Figure 4 is the comparison of intensities by the identified mycotoxin markers, in which the intensities were averaged by different class of samples. It can be observed that most of the exogenous marker compounds were in small amounts. In fact, 265 out of all 379 markers were even not present in normal wheats. In comparison, 368 out of 379 identified markers were presented in the moldy samples, most of them were in large amounts. Figure 5 is the PCA scores plot of wheat samples by mycotoxin markers only. The class distribution and degrees of separation were consistent with previous modelling in Figure 2. The PLS-DA result (data not shown) also showed no significant differences between the complete data set from the selected subsets by mycotoxin markers. Although the identification of mycotoxin markers was preliminary and pending further investigation such as multiple reaction monitoring (MRM) by LC–MS/MS with reference standards, the results indicated suitability of UPLC-QTOF-MS for large-scale marker screening. Furthermore, the large number of mycotoxins identified by UPLC-QTOF-MS indicated that quantification with a limited amount of toxins may not be suitable for wheat contamination due to the complicate variety of exogenous substances. Other peaks were remained to be unidentified and future studies based on in-depth database searching and experimental validation is still needed. Nevertheless, the non-targeted metabolomics can provide useful evidence for the selection of candidate markers for further development of rapid targeted screening approach.

## 4. Conclusion

The research successfully detected the simulated moldy wheat samples based on a non-targeted metabolomics with the established chemical information database of endogenous small molecules in wheat. The moldy wheat samples possibly contaminated by fungi were prepared, and compared with their normal counterparts. The differences between these two sample classes were characterized preliminary study by visual appearances and PCA modelling, followed by supervised PLS-DA. While visual identification depends on human experience and PCA could only partly identify the moldy wheat from the LCMS spectra, the PLS-DA were successfully used to distinguish them from normal wheat both in the scores plot and in a fully automated fashion. The results proved the non-targeted modelling advantages in processing a complex data set collected from high-precision analytical instruments such as from the high-resolution mass spectrometry. The PLS loading reflect plenty of information that related to compositional changes during the mildew process, as demonstrated by the corresponding loadings of both 59 endogenous and 379 exogenous potential marker compounds. The result showed that the non-targeted approach combined with high-resolution mass spectrometry is advantageous for foodborne microbial contamination to rapidly identify a large number of potential marker compounds without the need of certain standards. Particularly, mycotoxin markers presented positive changes toward moldy samples, which may be further used as candidates for application-specific rapid targeted screening. To a greater extent, in-depth research on such potential hazard factors would help to find and identify trace metabolites of fungal infection in wheat, and laid a foundation for the subsequent construction of typical markers of wheat fungal infection.

## Data availability statement

The raw data supporting the conclusions of this article will be made available by the authors, without undue reservation.

## Author contributions

BG: conceptualization and funding acquisition. MJ: methodology and investigation. WL: validation and writing—review and editing. MJ and YC: writing—original draft preparation. All authors contributed to the article and approved the submitted version.

## Funding

This research was funded by The National Natural Science Foundation of China (grant nos. 32001819 and 32272426).

## Conflict of interest

The authors declare that the research was conducted in the absence of any commercial or financial relationships that could be construed as a potential conflict of interest.

## Publisher’s note

All claims expressed in this article are solely those of the authors and do not necessarily represent those of their affiliated organizations, or those of the publisher, the editors and the reviewers. Any product that may be evaluated in this article, or claim that may be made by its manufacturer, is not guaranteed or endorsed by the publisher.

## References

[ref1] Cifuentes A. (2009). Food analysis and Foodomics foreword. J. Chromatogr. A 1216:7109. doi: 10.1016/j.chroma.2009.09.018 19765718

[ref2] Cirio M. Villarreal M. Seal T. M. L. Simon M. E. Smersu C. S. S. Kneeteman E. A. (2019). Incidence of deoxynivalenol in wheat flour in Argentina and GC-ECD method validation. J. AOAC Int. 102, 1721–1724. doi: 10.5740/jaoacint.19-0029, PMID: 31076023

[ref3] Cordewener J. H. G. Luykx D. M. A. M. Frankhuizen R. Bremer M. G. E. G. Hooijerink H. America A. H. P. (2009). Untargeted LC-Q-TOF mass spectrometry method for the detection of adulterations in skimmed-milk powder. J. Sep. Sci. 32, 1216–1223. doi: 10.1002/jssc.200800568, PMID: 19301324

[ref4] Creydt M. Fischer M. (2018). Omics approaches for food authentication. Electrophoresis 39, 1569–1581. doi: 10.1002/elps.201800004, PMID: 29572870

[ref5] Dong X. Liu C. Wu S. (2012). Research progress of insecurity determination wheat flour. J. Beijing Technol. Bus. Univ. (in Chinese). 30, 80–84.

[ref6] Femenias A. Gatius F. Ramos A. J. Sanchis V. Marin S. (2020). Use of hyperspectral imaging as a tool for Fusarium and deoxynivalenol risk management in cereals: a review. Food Control 108:106819. doi: 10.1016/j.foodcont.2019.106819

[ref7] Gab-Allah M. A. Lijalem Y. G. Yu H. Lee S. Baek S. Y. Han J.. (2023). Development of a certified reference material for the accurate determination of type B trichothecenes in corn. Food Chem. 404:134542. doi: 10.1016/j.foodchem.2022.134542, PMID: 36244066

[ref8] Gao B. Holroyd S. E. Moore J. C. Laurvick K. Gendel S. M. Xie Z. (2019). Opportunities and challenges using non-targeted methods for food fraud detection. J. Agric. Food Chem. 67, 8425–8430. doi: 10.1021/acs.jafc.9b03085, PMID: 31322874

[ref9] Gu X. L. Guo W. Y. Zhao Y. J. Liu G. Wu J. N. Chang C. (2019). Deoxynivalenol-induced cytotoxicity and apoptosis in IPEC-J2 cells through the activation of autophagy by inhibiting PI3K-AKT-mTOR signaling pathway. ACS Omega 4, 18478–18486. doi: 10.1021/acsomega.9b03208, PMID: 31720552 PMC6844115

[ref10] Hakme E. Lozano A. Gomez-Ramos M. M. Hernando M. D. Fernandez-Alba A. R. (2017). Non-targeted evaluation of contaminants in honey bees and pollen samples by gas chromatography time-of-flight mass spectrometry. Chemosphere 184, 1310–1319. doi: 10.1016/j.chemosphere.2017.06.089, PMID: 28679151

[ref11] He X. Wang R. Liu L. Guo Y. (2019). Study on mildew critical point and mildew rule of stored wheat. Cereals Oils. (in Chinese). 32, 84–88.

[ref12] Herrero M. Simo C. Garcia-Canas V. Ibanez E. Cifuentes A. (2012). Foodomics: MS-based strategies in modern food science and nutrition. Mass Spectrom. Rev. 31, 49–69. doi: 10.1002/mas.20335, PMID: 21374694

[ref13] Huang S. Cai J. Tian H. (2010). Mould development characters of different stored grains. J. Chin. Cereals Oils Assoc. (in Chinese). 25, 99–127.

[ref14] Jin M. Zheng W. Zhang Y. Gao B. Yu L. (2021). Lipid compositions and geographical discrimination of 94 geographically authentic wheat samples based on UPLC-MS with non-targeted lipidomic approach. Foods 10:10. doi: 10.3390/foods10010010, PMID: 33374499 PMC7822159

[ref15] KesKin E. Eyupoglu O. E. (2023). Determination of mycotoxins by HPLC, LC-MS/MS and health risk assessment of the mycotoxins in bee products of Turkey. Food Chem. 400:134086. doi: 10.1016/j.foodchem.2022.134086, PMID: 36075166

[ref16] Kunzelmann M. Winter M. Aberg M. Hellenas K. E. Rosen J. (2018). Non-targeted analysis of unexpected food contaminants using LC-HRMS. Anal. Bioanal. Chem. 410, 5593–5602. doi: 10.1007/s00216-018-1028-4, PMID: 29594430 PMC6096699

[ref17] Li F. Jiang D. Zhou J. Chen J. Li W. Zheng F. (2016). Mycotoxins in wheat flour and intake assessment in Shandong province of China. Food Addit. Contam. Part B-Surveill. 9, 170–175. doi: 10.1080/19393210.2016.1154109, PMID: 26892316

[ref18] Li B. Li G. Liu Q. (2003). Investigation of microorganism contamination on wheat and its by-products. Grain Storage. (in Chinese). 32, 36–38.

[ref19] Ramadhaningtyas D. P. Aryana N. Aristiawan Y. Styarini D. (2017). “Optimization of chromatographic conditions for determination of aflatoxin B1, B2, G1 and G2 by using liquid chromatography-mass spectrometry, in proceedings of the 3rd international symposium on applied chemistry” in AIP Conference Proceedings. eds. Tursiloadi S. Rinaldi N. (Melville: Amer Inst Physics).

[ref20] Rana N. Rahim M. S. Kaur G. Bansal R. Kumawat S. Roy J.. (2020). Applications and challenges for efficient exploration of omics interventions for the enhancement of nutritional quality in rice (Oryza sativa L.). Crit. Rev. Food Sci. Nutr. 60, 3304–3320. doi: 10.1080/10408398.2019.1685454, PMID: 31718237

[ref21] Righetti L. Rubert J. Galaverna G. Folloni S. Ranieri R. Stranska-Zachariasova M.. (2016). Characterization and discrimination of ancient grains: a metabolomics approach. Int. J. Mol. Sci. 17:1217. doi: 10.3390/ijms17081217, PMID: 27472322 PMC5000615

[ref22] Röst H. Sachsenberg T. Aiche S. Bielow C. Weisser H. Aicheler F.. (2016). OpenMS: a flexible open-source software platform for mass spectrometry data analysis. Nat. Methods 13, 741–748. doi: 10.1038/nmeth.3959, PMID: 27575624

[ref23] Sabillon L. Bianchini A. (2016). From field to table: a review on the microbiological quality and safety of wheat-based products. Cereal Chem. 93, 105–115. doi: 10.1094/cchem-06-15-0126-rw

[ref24] Sulyok M. Stadler D. Steiner D. Krska R. (2020). Validation of an LC-MS/MS-based dilute-and-shoot approach for the quantification of > 500 mycotoxins and other secondary metabolites in food crops: challenges and solutions. Anal. Bioanal. Chem. 412, 2607–2620. doi: 10.1007/s00216-020-02489-9, PMID: 32078002 PMC7136310

[ref25] Sun H. Lu W. Gao B. (2021). Non-targeted detection of butter adulteration using pointwise UHPLC-ELSD and UHPLC-UV fingerprints with chemometrics. Food Chem. 356:129604. doi: 10.1016/j.foodchem.2021.129604, PMID: 33819790

[ref26] Vidal J. C. Bertolin J. R. Ezquerra A. Hernandez S. Castillo J. R. (2017). Rapid simultaneous extraction and magnetic particle-based enzyme immunoassay for the parallel determination of ochratoxin a, fumonisin B1 and deoxynivalenol mycotoxins in cereal samples. Anal. Methods 9, 3602–3611. doi: 10.1039/c7ay00386b

[ref27] Wei T. Ren P. P. Huang L. L. Ouyang Z. C. Wang Z. Y. Kong X. F.. (2019). Simultaneous detection of aflatoxin B1, ochratoxin a, zearalenone and deoxynivalenol in corn and wheat using surface plasmon resonance. Food Chem. 300:125176. doi: 10.1016/j.foodchem.2019.125176, PMID: 31351258

[ref28] Yu L. Lu W. Liu J. Du L. (2016). Review on non-targeted detection technique for food safety and quality. J. Food Sci. Technol. 34, 1–6. doi: 10.3969/j.issn.2095-6002.2016.06.001

[ref29] Zhou J. Peng X. Gao Y. Song J. Jiang T. Ju X.. (2010). Effects of temperature and relative humidity on mould floras in wheat flour stored. J. Chin. Cereals Oils Assoc. (in Chinese). 25, 94–97.

